# Comparative genome analysis of jujube witches’-broom Phytoplasma, an obligate pathogen that causes jujube witches’-broom disease

**DOI:** 10.1186/s12864-018-5075-1

**Published:** 2018-09-19

**Authors:** Jie Wang, Laiqing Song, Qiqing Jiao, Shuke Yang, Rui Gao, Xingbo Lu, Guangfang Zhou

**Affiliations:** 1 Shandong Institute of Pomology, Taian, 271000 China; 2 Yantai Academy of Agricultural Sciences, Yantai, 265500 China; 3 Institute of Plant Protection, Shandong Academy of Agricultural Sciences, Jinan, 250100 Shandong China

**Keywords:** Phytoplasma, Genome sequence, Synteny, Potential mobile unit (PMU)

## Abstract

**Background:**

JWB phytoplasma is a kind of insect-transmitted and uncultivable bacterial plant pathogen causeing a destructive Jujube disease. To date, no genome information about JWB phytoplasma has been published, which hindered its characterization at genomic level. To understand its pathogenicity and ecology, the genome of a JWB phytoplasma isolate jwb-nky was sequenced and compared with other phytoplasmas enabled us to explore the mechanisms of genomic rearrangement.

**Results:**

The complete genome sequence of JWB phytoplasma (jwb-nky) was determined, which consisting of one circular chromosome of 750,803 bp with a GC content of 23.3%. 694 protein-encoding genes, 2 operons for rRNA genes and 31 tRNA genes as well as 4 potential mobile units (PMUs) containing clusters of DNA repeats were identified. Based on PHIbaes analysis, a large number of genes were genome-specific and approximately 13% of JWB phytoplasma genes were predicted to be associated with virulence. Although transporters for maltose, dipeptides/oligopeptides, spermidine/putrescine, cobalt, Mn/Zn and methionine were identified, KEGG pathway analysis revealed the reduced metabolic capabilities of JWB phytoplasma. Comparative genome analyses between JWB phytoplasma and other phytoplasmas shows the occurrence of large-scale gene rearrangements. The low synteny with other phytoplasmas indicated that the expansion of multiple gene families/duplication probably occurred separately after differentiation.

**Conclusions:**

In this study, the complete genome sequence of a JWB phytoplasma isolate jwb-nky that causing JWB disease was reported for the first time and a number of species-specific genes were identified in the genome. The study enhanced our understandings about genomic basis and the pathogenicity mechanism of this pathogen, which will aid in the development of improved strategies for efficient management of JWB diseases.

**Electronic supplementary material:**

The online version of this article (10.1186/s12864-018-5075-1) contains supplementary material, which is available to authorized users.

## Background

Pytoplasma was a group of plant pathogenic bacteria that belong to the class of *Mollicutes*. Phylogenetic analysis, virtual restriction fragment length polymorphism (RFLP) and online software i*PhyClassifier* analysis of 16S rRNA gene sequences have become the widely accepted phytoplasma classification schemes [1]. Pytoplasmas can affect a wide range of plant hosts, including agriculturally and economically important plants, such as fruit tree, landscape plant and flowers. Phytoplasma infected plants showing various symptoms ranging from mild yellowing to death. As wall-less obligate parasites, phytoplasma propagate intracellularly in both insects and plant phloem and transmit from plant to plant by insect vectors [2]. Although axenic cultivation of phytoplasmas was reported [3], most of the phytoplasma strains are not successfully cultivated in vitro owe to the strict anaerobic conditions and complex media.

Whole-genome projects can provide insight into the organism biology and help understand host-pathogen interactions. However, the difficult cultivated in vitro of phytoplasmas poses a challenge to the collection of sufficiently high-quality phytoplasma genomic DNA and hinders attempts to study structural features and genome organization. Thus far, only six complete phytoplasma genomes have been fully sequenced [4–9], in which, three from 16Sr I group phytoplasmas (yellows mild strain OY-M, aster yellows witches’-broom isolate AY-WB and maize bushy stunt phytoplasma MBSP), two from 16SrXII phytoplasmas (Australian isolate PAa and strawberry lethal yellows isolate SLY of ‘*Candidatus* Phytoplasma australiense’) and one from 16Sr X phytoplasma (apple proliferation phytoplasma AT of ‘*Ca*. *P. mali*’). In addition, fifteen partial draft genomes, including members of 16SrI, 16SrII, 16SrIII, 16SrIX, 16SrXI and 16SrXII groups, have been reported [10–21]. Based on these sequences, more and more genomic information of phytoplasma were obtained. The genome length of phytoplasmas is between 530 kb and 1350 kb, and the size of the closely related strains varies significantly [22]. The genomes have a relatively low GC content (23.0–29.5 Mol%), two rRNA operons, a small amount of tRNAs and a limited set of genes encoding metabolic enzymes [5].

Chinese Jujube (*Ziziphus jujuba* Mill.), a native and economically important fruit tree of China, is one of the most important members in the *Rhamnaceae* family. The complex jujube genome and more and more function genes were identified [23–25]. Jujube witches’-broom (JWB) disease caused by JWB phytoplasma widespread in China and causes serious problems to the industry. Until the phytoplasmal cells were demonstrated through transmission electron microscopy [26], JWB disease was described as a graft-transmissible viral disease [27]. JWB phytoplasma now classified as ‘*Ca. P. ziziphi*’, belongs to elm yellows group (16SrV) subgroup B based on analyses of 16S rDNA sequences [28, 29]. The infected plants exhibit a variety of symptoms, such as little leaves, phyllody, witches’ broom, leaves etiolating and eventually death within a few years of infection [30]. Previous studies mainly focused on the investigations of JWB, whereas very little knowledge is known about its pathogenicity mechanisms. Some progresses have been made in understanding the regulation of plant-phytoplasma interactions, and several candidate genes and proteins that might be involved in the interaction of Mexican lime trees with the phytoplasma were identified [31, 32]. Recently, microRNAs that may participate in the JWB phytoplasma response of jujube have been identified [33]. Furthermore, combination of iTRAQ proteomics and RNA-seq transcriptomics reveals multiple levels of regulation in phytoplasma-infected jujube [34]. However, the molecular mechanism of JWB disease is not fully understood. In this study, the complete genome sequence of JWB phytoplasma was reported, as well as and the relationship with other five phytoplasmas and the potential key genes were determined by comparative gene analysis. This work is of great importance for a comprehensive understanding of the interactions between JWB phytoplasmas and their hosts.

## Methods

### Phytoplasma sources

Samples of *Z. jujuba* (Jujube) with witches’-broom were collected from Shandong, China. The phytoplasma strain was then maintained and propagated in an insect-proof glasshouse by tissue culture. The transmitted JWB phytoplasma strain was confirmed by PCR using non-specific primers 16S rDNA-F (5’-TAAAAAGGCATCTTTTTGTT-3′) and 16S rDNA-R (5’-AATCCGGACTAAGACTGT-3′) that amplify the 16S rDNA sequence [28].

### Genome DNA preparation and whole genome sequencing

JWB phytoplasma chromosomal DNA was prepared as described previously [12]. JWB phytoplasma pellets were purified by differential centrifugation. Phytoplasma DNA were separated by pulsed-field gel electrophoresis (PFGE) after digestion by proteinase K at 50 °C for 72 h. Yeast chromosomes (New England Biolabs, Frankfurt, Germany) were used as a molecular size marker. The DNA was then electroeluted from the excised PFGE agars slice and concentrated by ethanol precipitation. To improve the accuracy of the genome sequences, Illumina HiSeq 4000 platform and PacBio RS II platform (10 kb inserts library; Pacific Biosciences, Menlo Park, CA, USA) were used for the JWB-nky sequencing at the Beijing Genomics Institute (BGI, Shenzhen, China). Four SMRT cells Zero-Mode Waveguide arrays of sequencing were used by the PacBio platform to generate the subreads set. PacBio subreads (length < 1 kb) were removed.

### Genome assembly and annotation

PacBio sequencing reads were de novo assembled with hierarchical genome-assembly process (HGAP) [35]. Sequencing data were combined, and further polished with Pion [36] to obtain the complete sequences. Removed genomic gene of plant based on the whole jujube genome sequence [23]. Prodigal v2.60 [37] was used to predict open reading frames (ORFs) for the JWB phytoplasma genome sequence. UGA was used as a stop codon, which was consistent with the ORF prediction for other phytoplasmas. tRNAscan-SE was applied to identify tRNAs [38] and RNAmmer [39] was used to predict the locations of rRNA genes. JWB phytoplasma genes were named according to their homologous genes and OrthoMCL [40] was used to identify homologous genes between the JWB phytoplasma genome and other complete phytoplasma genomes. Similarity searches were carried out using BLASTP [41] against the UniProt database. Those genes with no homology to the other complete phytoplasma genomes were searched by BLASTP against the NCBI nr database. The rest of the genes, which had no homology with the phytoplasma complete genomes or the nr database, were presumed to be putative protein-coding genes only when they were longer than 100 amino acids or had a confidence score of more than 10 from Prodigal. COG (Clusters of Orthologous Groups) [42], KEGG (Kyoto Encyclopedia of Genes and Genomes, http://www.genome.jp/kegg/), NR (Non-Redundant Protein Database) and GO (Gene Ontology) databases were used for general function annotation. The program signalP V4.0 [43] was used to predict SP cleavage in all proteins. For SP-containing proteins, TMHMM V2.0 [44] was used to predict the transmembrane regions after eliminating the SP. Virulence factors and resistance gene were identified based on the core dataset in VFDB (Virulence Factors of Pathogenic Bacteria) database, PHI (Pathogen Host Interactions) database and CAZy (Carbohydrate-Active enZYmes Database).

### Comparative genome analysis

For comparative genomic analysis, five complete phytoplasma genomes (*Ca.P. australiense* [‘Ca. P. australiense’, GenBank accession number: GCF_000069925.1], *Candidatus* Phytoplasma mali [‘Ca. *P. mali*’, GenBank accession number: GCF_000026205.1], *Candidatus* Phytoplasma pruni [‘Ca. *P. pruni*’, GenBank accession number: GCA_001277135.1], Onion yellows phytoplasma [OY-M, GenBank accession number: GCA_000009845.1], Peanut witches’-broom phytoplasma [PnWB NTU2011, GenBank accession number: GCA_000364425.1]) were chosen for comparison. OrthoMCL was used for gene clustering analysis. All genes conserved among the six phytoplasma genomes were aligned using MUSCLE [45] with the default settings. The resulting multiple sequence alignment was used to infer the species phylogeny using the maximum likelihood program PhyML [46]. The visualization of syntenic blocks was done in Web-based Genome Synteny Viewer [47]. BLASTP was used for the alignment of protein sequences.

### Nucleotide sequence accession number

The JWB phytoplasma chromosome sequence was deposited in DDBJ/EMBL/GenBank nucleotide sequence databases under accession number CP025121.

## Results and discussion

### General features of the JWB phytoplasma genome

**Fig. 1:**
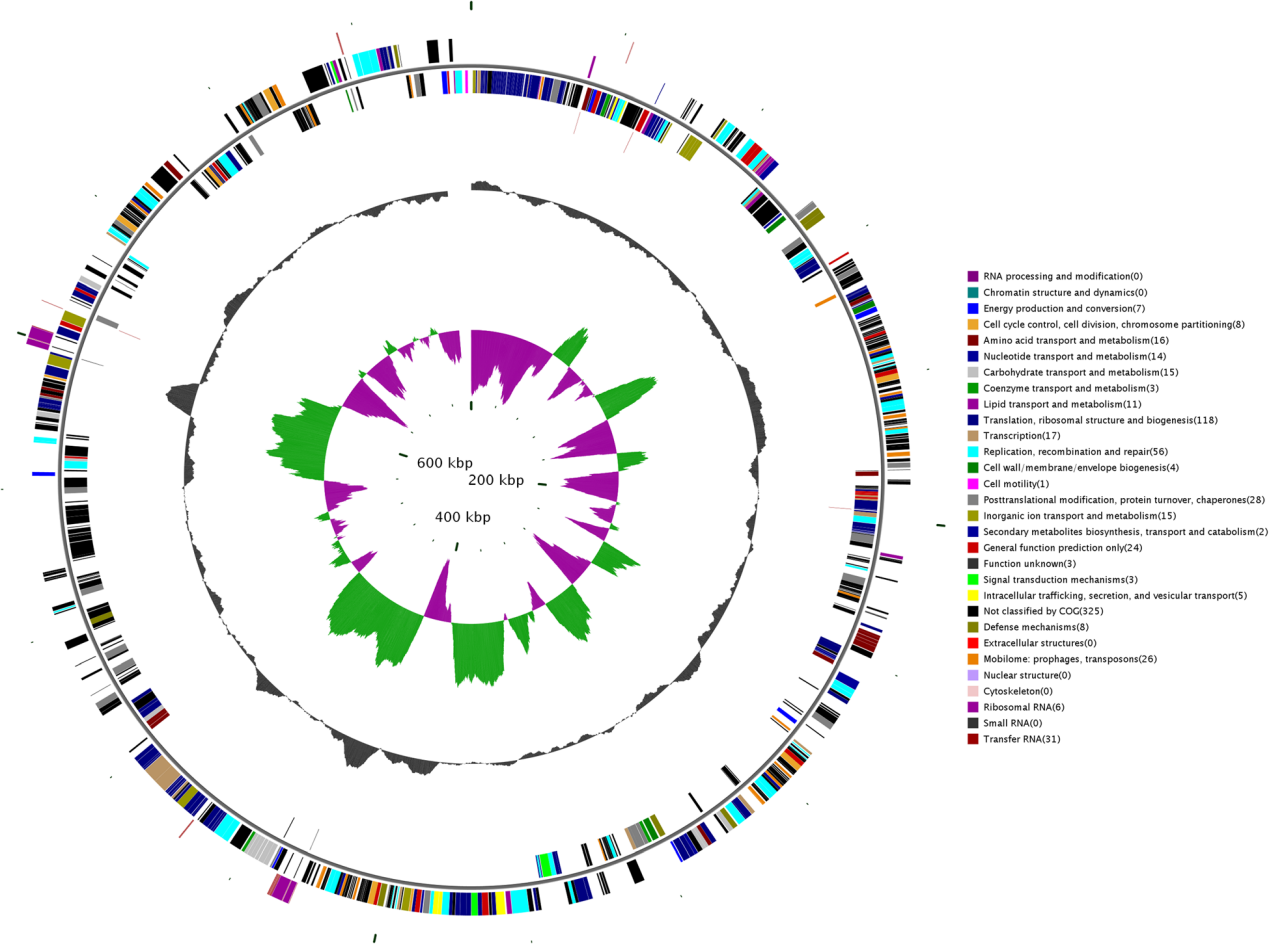
Genome map of the 750,803-bp circular chromosome of JWB phytoplasma jwb-nky. Rings from the outside to inside are as follows: ring 1, predicted ORFs on the sense strand; ring 2, predicted ORFs on the antisense strand; ring 3, fragmented genes on the sense strand; ring 4, fragmented genes on the antisense strand; ring 5, locations of rRNA genes (brown), tRNA genes (gray), and miscellaneous RNAs (black); ring 6, PMUs on the sense strand; ring 7, PMUs on the antisense strand

**Fig. 2:**
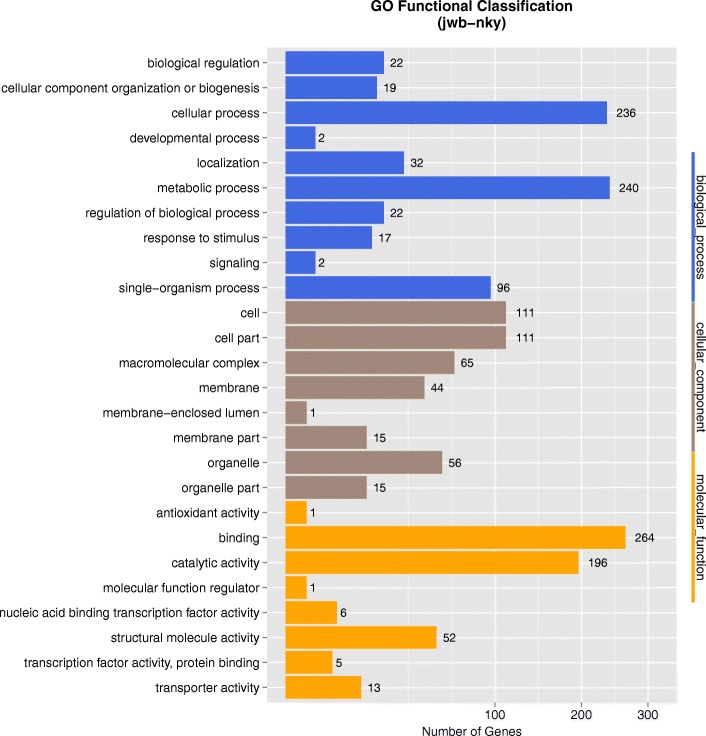
Gene Ontology categories of Phytoplasma according to function

**Fig. 3:**
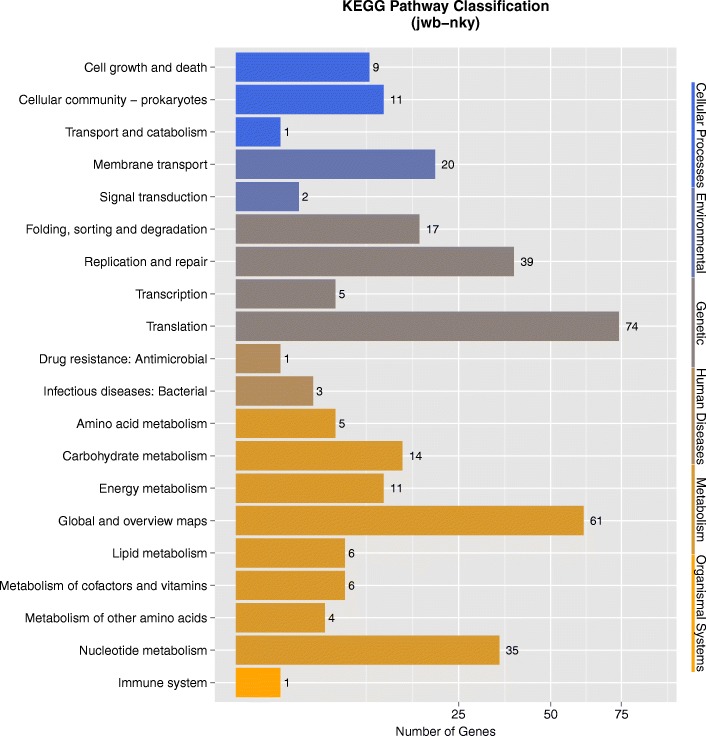
KEGG pathway analysis of Phytoplasma

**Fig. 4:**
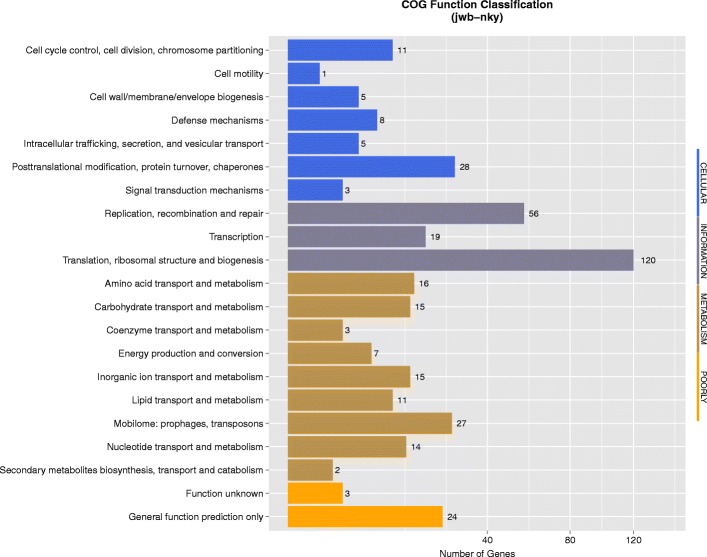
The COG functional category of JWB phytoplasma genes

The JWB phytoplasma genome consists of 750,803 bp with GC content of 23.31%. Based on the annotation, 694 protein-coding genes, two operons for rRNA genes and 31 tRNA genes were found in the genome (Table 1, Fig. 1). Genes in jwb-nky were annotated with different databases (See Additional file 1). 513 of the 694 annotated protein coding genes in jwb-nky can be annotated to Non-Redundant Protein Database based on homologous alignment with E-value< 10^− 5^. The genes homologous to Ca. Phytoplasma are the most abundant, accounting for 38.52% of total genes. 377 genes were categorized into cellular components, molecular functions, and biological processes according to GO (gene ontology) analysis (Fig. 2, Table 2). The most predominant cellular components were located in the cell (111) and cell part (111). The main molecular functions were binding activity (264) and catalytic activity (196). The most common biological processes were metabolic process (240), cellular process (236) and single-organism process (96). Other major functional categories were membrane (44), macromelocular complex (65), orgenalle (56) and structural molecular activity (52). Pathway analysis categorized 328 genes into 4 levels (Fig. 3, Table 2). It is worth noting that most genes in metabolism were associated with global and overview maps (61), carbohydrate metabolism (14) and energy metabolism (11); in genetic information processing were replication and repair (39) and translation (74); and in environmental information processing were membrane transport (20). 369 genes had specific functional ditributions according to the COG categories (Fig. 4, Table 2) and these 369 genes were assigned to 19 functional categories. The most abundant functional category was COG category J (translation, ribosomal structure and biogenesis) (120), accounting for 17.29% of the JWB genes, mainly due to 2 ribosomal proteins and 31 tRNA synthetases found in the genome. Followed by the COG category L (replication, recombination and repair), which contained 56 genes. A total of 537 protein-coding genes (77.37% of protein coding genes) were annotated to NR/GO/KEGG/COG database (See Additional file 1). As described in “*Ca*. *P. asteris*” strains OY-M [4]and AY-WB [5]and Ca. P. australiense [6], UGA was also used as a stop codon for ORF prediction.

**Table 1:** Genome assembly statistics

Characteristic	Value for group					
	jwb-nky	‘Ca. P. australiense’	‘Ca. *P. mali*’	‘Ca. P. runi’	OY-M	PnWB NTU2011
Length (bp)	750,803	879,959	601,943	598,511	853,092	562,473
G + C content (%)	23.3	27.4	21.4	27.2	27.8	24.3
No. of protein coding genes	694	684	479	550	749	421
Protein-coding regions (%)	77.7	64.1	76.1	xx	72.8	68.2
No. of tRNA genes	31	35	32	xx	32	27
No. of rRNA operons	2	2	2	2	2	2

About XX in ‘Ca. *P. pruni*’, the data were sourced from the published literatures, and no statistics on only the announcements in the literature

**Table 2:** The number of genes annotated in different database

Species	NR	GO	KEGG	COG	All
jwb-nky	513	377	328	369	537
Candidatus Phytoplasma australiense	649	411	327	374	650
Candidatus Phytoplasma mali	460	319	288	310	460
Candidatus Phytoplasma pruni	442	250	289	300	442
Onion yellows phytoplasma	708	416	317	393	709
Peanut witches’-broom phytoplasma	429	246	263	287	429

### Comparative genomic analysis of phytoplasmas

#### Clustering of gene family and core gene analysis

**Fig. 5:**
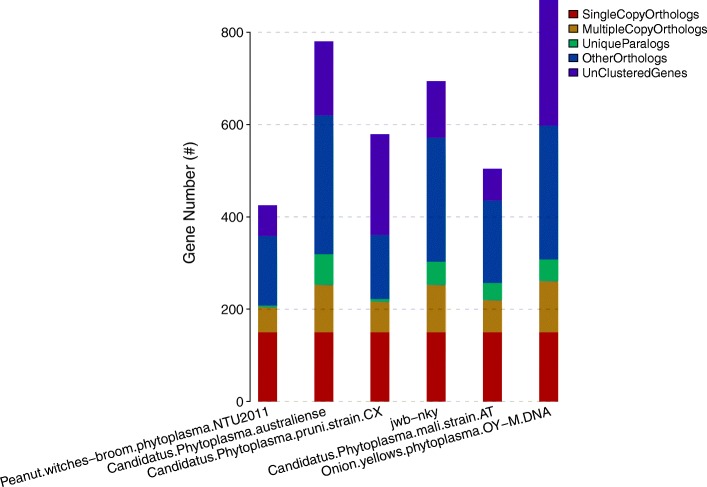
The number of clustering genes from 6 different phytopathogens

JWB phytoplasma has more multiple-copy orthologs with Ca. P. australiense and OY-M, and the genome size of these phytoplasmas is larger than that of Ca. *P. mali*, Ca. *P. pruni* and PnWB (Fig. 5), suggesting that expansion of multiple gene families/duplication occurred in these genomes and may play an important role in pathogenesis.

70 core genes were identified in all of these genomes: 12 from JWB, 14 from ‘*Ca*. P. australiense’, 9 from Ca. *P. mali*, 12 from Ca. *P. pruni*, 9 from OY-M and 14 from PnWB (See Additional file 2). In addition to these homologous genes, JWB phytoplasma also contained 307 specific genes, with no assigned function.

#### Phylogenetic relationships

**Fig. 6:**
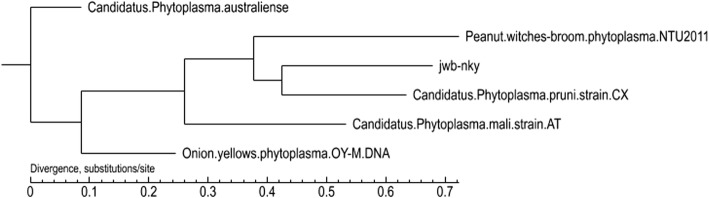
Phylogeny of phytoplasmas. The organismal phylogeny based on the concatenated alignment of 150 single-copy genes conserved among the six phytoplasma genomes

A total of 150 single copy gene proteins, common to all 6 phytoplasma genomes were identified (see Additional file 3). The phylogenetic tree based on the concatenated alignment of these genes showed that JWB phytoplasma were grouped closely with Ca. *P. pruni* and remotely with Ca. P. australiense (Fig. 6), indicating that the expansion of multiple gene families/duplication occurred separately after divergence.

#### Gene synteny

**Fig. 7:**
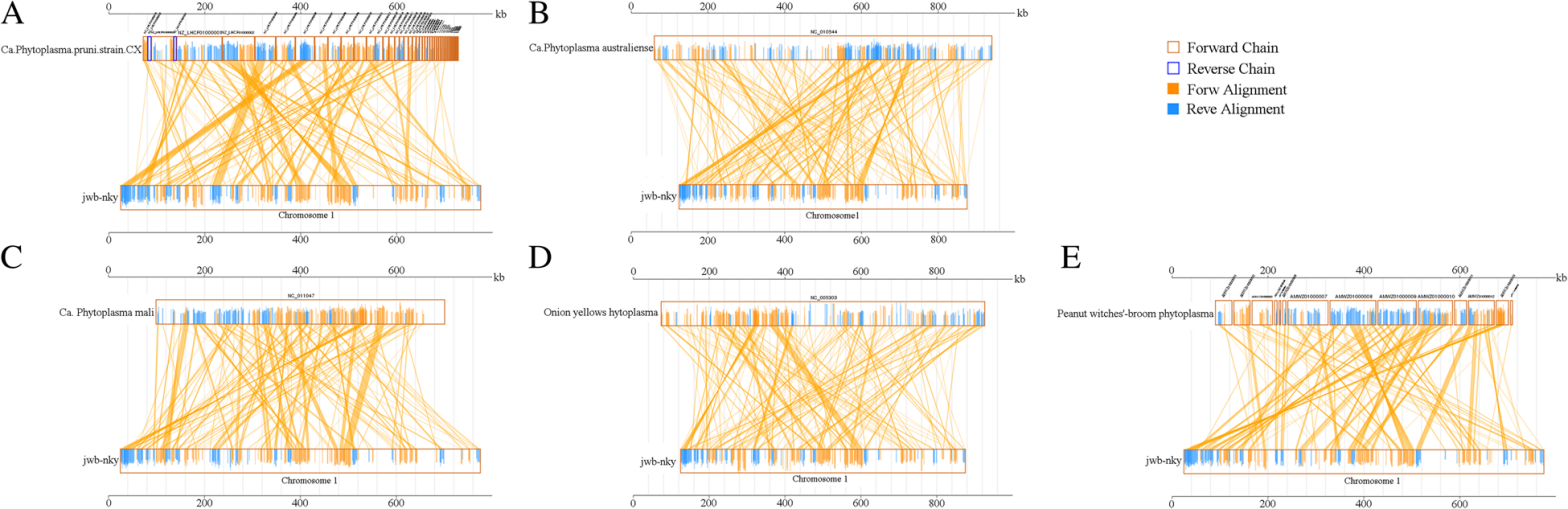
Whole-genome alignment reveal the gene synteny. The chromosomes are shown in linearized form to illustrate relative gene synteny A,B,C,D,E indicated the alignment of JWB with with Ca. Phytoplasma pruni (**a**), Ca.Phytoplasma australiense (**b**), Ca. Phytoplasma mali (**c**), Onion yellows hytoplasma (**d**) and Peanut witches’-broom phytoplasma (**e**) respectively

Genomic synteny analysis between the JWB phytoplasma genome and other complete phytoplasma genomes were performed. Synteny between these genomes is quite poor. The alignment among the genomes of JWB phytoplasma, Ca. P. australiense, Ca. *P. mali*, OY-M and PnWB showed some of synteny regions (Fig. 7b, c, d, e). However, the genome alignments between JWB and Ca. *P. pruni* showed more synteny regions than other phytoplasmas (Fig. 7a). The result from synteny analysis is consistant with the phylogenetic relationships in which JWB and Ca. *P. pruni* were in the same group (Fig. 6).

#### Metabolic pathway

The metabolic capabilities of many pathways are greately reduced in phytoplasmas, as is in the JWB phytoplasma. Investigation of the genome sequences showed that the pathways for oxidative phosphorylation, amino acid and fatty acid biosynthesis, pentose phosphate pathway and tricarboxylic acid cycle are not completed in JWB phytoplasma. It lacks genes involved in most amino acid sythesis, and only one gene involved in the reversible conversions of L-serine to glycine (jwb-nkyGM000266) was identified. And as is in other phytoplasmas, the pathways for ATP synthesis and fatty acid metabolism in JWB phytoplasma are incomplete. Only one gene involved in oxidative phosphorylation (inorganic pyrophosphatase, jwb-nkyGM000439) and seven genes related to glycerolipid and glycerophospholipid metabolism (*gspA*, jwb-nkyGM000052; *plsY*, jwb-nkyGM000048, *pssA*, jwb-nkyGM000102; *psd*, jwb-nkyGM000103; *plsX*, jwb-nkyGM000108; *acpS*, jwb-nkyGM000692 and *cdsA*, jwb-nkyGM000069) were identified. It also lacks a vitamin B_6_ metabolism gene (*pdxK*) and folate biosynthesis genes (*folKPC*), which were found in the OY-M phytoplasma genome.

None of the ATP-synthase subunits were identified in the JWB phytoplasma genome, while genes involved in glycolysis were detected (*pgi*, jwb-nkyGM000319; *pfkA*, jwb-nkyGM000444; *fba*, jwb-nkyGM000447; *gapA*, jwb-nkyGM000446; *pgk*, jwb-nkyGM000445; *gpmI*, jwb-nkyGM000443; *eno*, jwb-nkyGM000442; *pykF*, jwb-nkyGM000551; *tpiA*, jwb-nkyGM000540). suggesting that JWB phytoplasma depends on glycolysis for its energy generation. The phosphoenol pyruvate dependent sugar phosphotransferase (PTS) systems responsible for sugar importation and phosphorylation were not identified, whereas three genes encoding ABC-type maltose transporters (*malF*, jwb-nkyGM000324; *malG*, jwb-nkyGM000325; *malK*, jwb-nkyGM000323) were found in JWB phytoplasma. The maltose ABC transporter could also recognize sucrose and trehalose in some bacteria [48], which are major sugars in plant phloem and insect hemolymph, respectively. As in WBD phytoplasma [12], enzymes responsible for phosphorylation of glucose to produce glucose 6-phosphate were not identified in the JWB phytoplasma genome. Although a sucrose phosphorylase (jwb-nkyGM000581) catalyzing the conversion of sucrose to D-fructose and *α*-D-glucose-1-phosphate was found, no phosphoglucomutase gene was identified. A putative malate/citrate symporter (*citS*, jwb-nkyGM000689) and a malate dehydrogenase (*sfcA*, jwb-nkyGM000280) were found in other metabolic pathways that enabled the JWB phytoplasma to use malate, and this carbon source for ATP generation in phytoplasmas [8]. No enzymes for the conversion of pyruvate to acetyl-CoA and acetyl-P/ADP to acetate/ATP were found. Although no phosphate acetyltransferase was found to catalyze acetyl-CoA to produce acetul-p, a putative phosphate propanoyltransferase (*pduL*, jwb-nkyGM000388) was identifiedthat may catalyze this reaction in phytoplasma.

#### Transporter and secretion systems

Phytoplasmas have strongly reduced metabolic capabilities, so they must absorb metabolites from their plant and insect hosts. The JWB phytoplasma genome contains 13 ORFs that encode transport and 264 ORFs encode binding proteins. Two ABC transporter genes (*evbG*, jwb-nkyGM000115 and *mdlA*, jwb-nkyGM000116) involved in multidrug resistance were identified. Others including ATP-binding cassette (ABC) transporter systems for dipeptides/oligopeptides (*oppA*, jwb-nkyGM000118; *dppB*, jwb-nkyGM000257 and *dppD*, jwb-nkyGM000255), spermidine/putrescine (*potA*, jwb-nkyGM000481; *potB*, jwb-nkyGM000480; *potC*, jwb-nkyGM000479; *potD*, jwb-nkyGM000478), cobalt (*cbiO*, jwb-nkyGM000001), Mn/Zn (*znuB1*, jwb-nkyGM000084; *znuB2*, jwb-nkyGM000085; *znuC*, jwb-nkyGM000086) and D-methionine (*metN*, jwb-nkyGM000558, *metQ*, jwb-nkyGM000559, *metI* jwb-nkyGM000560) were identified. However, amino acid transporter systems except for D-methionine such as *GlnQ* and *ArtIMPQ* that are present in the AY strains [5] are lacking in JWB phytoplasma, which is similar to Ca. *P. mali* [8]. The methionine transport system may be also involved in other amino acids transportation.The components of protein translocation system were identified, including *secA* (jwb-nkyGM000372), *secY* (jwb-nkyGM000009), *yidC* (jwb-nkyGM000141), *ffh* (jwb-nkyGM000385), *ftsY* (jwb-nkyGM000384), *dnaJ* (jwb-nkyGM000334), *dnaK* (jwb-nkyGM000335), *grpE* (jwb-nkyGM000336), *groEL* (jwb-nkyGM000114) and signal peptidase *SPaseI* (jwb-nkyGM000063), which suggested that a functional *sec*-dependent protein translocation system exists in JWB phytoplasma.

#### Virulence-related factors

Plant-pathogenic bacteria can secrete a subset of carbohydrate-activated enzymes (CAZymes) to degrade plant cell walls. The JWB phytoplasma genome encodes three CAZymes: jwb-nkyGM000581 (GH13), jwb-nkyGM000537 (GH4) and jwb-nkyGM000495 (GT78) (See Additional file 4). jwb-nkyGM000537 and jwb-nkyGM000495 are specific to JWB phytoplasma, while jwb-nkyGM000581 has a homologue in ‘Ca. P. australiense’ with 70.37% identify. These enzymes may be involved in phytoplasma virulence.

Virulence factors such as hemolysins and adhesion-related proteins are thought to be involved in bacterial pathogenicity. JWB phytoplasma genome contains two genes encoding putative hemolysins (jwb-nkyGM000059, jwb-nkyGM000572), this is similar to the results of hemolysine related proteins identified in AY-WB phytoplasma and Ca. p. australiense. The virulence factor TENGU [49], effectors SAP11 [50] and SAP54 [51] have been shown to alter plant morphology. 28 candidate secreted JWB proteins (SAP) were identified according Bai et al. (data no show) [52]. 87 gene products in JWB phytoplasma have homologues in Virulence Factor Database (VFDB, http://www.mgc.ac.cn/VFs/main.htm) (See Additional file 5) and 92 gene products have homologues in Pathogen Host Interactions Database (PHI-base, http://www.phi-base.org/) using Blastp (E value< 10^− 5^) in which 13 gene products were in the class PHI:1566|GzOB006 and 11 genes were in the class PHI:2042|ABC3 (See Additional file 6). Among these factors, the SOD protein encoded by *sodA* (jwb-nkyGM000089), which was described as a virulence factor for *Mycoplasma pneumoniae* [53] was found. it has also been found in OY-W and WBD phytoplasma that SodA may play an important role in the phytoplasma colonization of plants and insects through degradation of reactive oxygen species (ROS) produced by the host during infection [54]. In addition, molecular chaperones DnaK and DnaJ have been reported to be necessary for pathogen survival in the host cell [55]. The multi-copy *hflB* (or *ftsH*) genes that encode membrane associated ATP-dependent Zn proteases also present in this group. These proteins are conserved among bacteria and are involved in protein secretion [56] and membrane protein assembly [57] as well as adaptations to nutritional conditions and osmotic stress [56, 58]. Other virulence-related factors in this group include AAA+ ATPases [59] and nucleases [60].

Besides virulence facotors, a large-conductance mechanosensitive channel MscL protein encoded by jwb-nkyGM000669 was identified. MscL involves in osmotic shock defense [61] and may help JWB phytoplasma adapt to changes in osmotic pressure between plant and insect cells. The presence of MscL, SodA, as well as DnaJ/DnaK suggests that JWB phytoplasma adapts to complex environments by different mechanisms.

#### PMUs

**Fig. 8:**
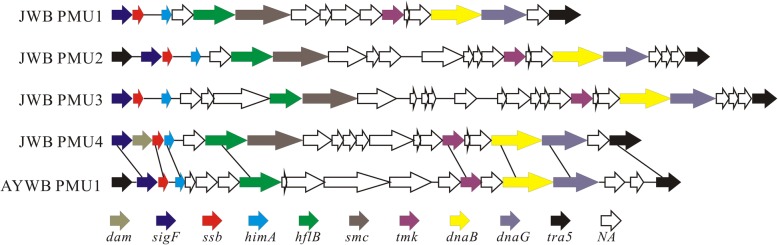
Potential mobile units (PMUs) from JWB phytoplasma and aster yellows-witches’ broom (AY-WB) phytoplasmas. Homologous genes between the PMUs are linked by lines. Dam, adenine-specifc DNA methyltransferase; sigF, RNA polymerase; sigma-F factor; ssb, single-stranded DNA-binding protein; himA, DNA binding protein HU; hflB, ATP-dependent Zn protease; smc, chromosome segregation ATPase-like protein; tmk, thymidylate kinase; dnaB, replicative DNA helicase; dnaG, DNA primase; tra5, putative transposase; NA, conserved hypothetical protein without assigned fuction

Genome sequencing has revealed that many phytoplasma genomes contain large repetitive regions called potential mobile units (PMUs) that appear to have promoted intrachromosomal recombination [5, 7]. PMUs contain several genes involved in gene duplication and transposition, and some contain putative ‘virulence genes’. Genome alignment suggests that PMUs are involved in the instability and recombination of phytoplasma genome. Four PMUs were identified in JWB phytoplasma genome (Fig. 8, Table 3) and they showed the highest similar structure with AY-WB PMU1. The phylogenetic tree based on PMUs gene support different relationships between the JWB PMUs and homologs in the PnWB PMU and ‘*Ca*. *P. asteris*’ OY-M and AY-WB genomes, suggesting that potential mobile units are transferred among these phytoplasmas. JWB PMUs are more likely to have been transferred from the 16SrI group, due to PnWB PMU is originated from one of the PMUs present in a close relative of 16SrI group OY-M [62]. PMUs translocation within the JWB genome resulted in the coexistence of different but homologous PMUs (PMU1–4), as in ‘*Ca*. P. australiense’ [6]. Due to the close association of effectors and PMUs [63], PMU-mediated horizontal gene transfers among different phytoplasmas may have an impact on the evolution of pathogenicity [62].

**Table 3:** The potential mobile units (PMUs) genes identifed in JWB phytoplasma genome

ORF	PMU1	PMU4	PMU2	PMU3	Product
2			tra5		putative transposase
3	SIG3.4	SIG3.4	SIG3.4	SIG3.4	DNA-directed RNA polymerase sigma-70 factor
4		dam			DNA methyltransferase
5	ssb	ssb	ssb	ssb	single-stranded DNA-binding protein
6	hupB	hupB	hupB	hupB	DNA-binding protein HU
7	NA	NA	NA	NA	hypothetical protein
8				NA	AAA+ ATPase
9				NA	group II intron reverse transcriptase
10	ftsH	hflB	ftsH	ftsH	ATP-dependent Zn protease
11	smc	smc	smc	smc	Chromosome segregation ATPase
12	NA	NA	NA	NA	hypothetical protein
13	NA	NA	NA, SAP06-like effector	NA SAP68-like effector	hypothetical protein
14	NA	NA effector protein		NA effector protein	hypothetical protein
15		NA SAP54-like effector		NA	hypothetical protein
16		NA	NA Phage-related protein	NA Phage-related protein	hypothetical protein
17		NA	NA	NA	hypothetical protein
18				NA	hypothetical protein
19			NA	NA	phage-associated protein
20			NA	NA	hypothetical protein
21	NA	NA	NA	NA	Phage-related protein
22	tmk	tmk,	tmk	tmk	thymidylate kinase
23	NA	NA	NA	NA	hypothetical protein
24	NA	NA	NA	NA	hypothetical protein
25	dnaB	dnaB	dnaB	dnaB	DNA helicase
26	dnaG	dnaG	dnaG	dnaG	DNA primase
27	NA outer membrane receptor proteins	NA outer membrane receptor proteins	NA	NA	hypothetical protein
28	tra5	tra5			putative transposase
29			NA	NA	hypothetical protein
30					hypothetical protein
31					DNA helicase
32			NA	NA	hypothetical protein
33			tra5	tra5	putative transposase
34			tra5	tra5	putative transposase

## Conclusion

Jujube witches’-broom (JWB) disease, caused by JWB phytoplasma, is one of the destructive diseases affecting worldwide jujube orchards. Some progresses have been made in understanding the regulation of Jujube-phytoplasma interactions. However, genomic basis and mechanism of its pathogenicity on Jujube remains to be researched. In this study, the genome of a JWB phytoplasma isolate jwb-nky was sequenced. Comparative genomic analysis revealed that, although jwb-nky is closely related to ‘*Ca*. *P. pruni*’ according to phylogenetic analysis, the gene syntety between the two phytoplasmas is low. And metabolic analysis suggested that some essential pathways of JWB phytoplasma are completely missing and others are greatly reduced.

Previous study based on 16S rDNA sequences analysis also indicates that the JWB phytoplasmas represents a novel taxon, ‘*Ca.* Phytoplasma ziziphi’ [28]. There is few report on genome sequence of other JWB phytoplasma isolates and the information about them are not sufficient to access the envolutionary relationships extensively. Therefore, in order to have a better understanding about the genomic basis, evolution and pathogenicity mechanism of this phytoplasmas group, more genomes of JWB phytoplasma isolates need to be sequenced in the future.

## Additional files

Additional file 1: Gene annotation of jwb-nky with different databases. (XLS 468 kb)

Additional file 2: The identity of core genes of the six phytoplasmas (XLS 6 kb)

Additional file 3: Single copy genes in six phytoplasmas. (XLS 41 kb)

Additional file 4: Annotated CAZymes in jwb-nky. (XLS 1 kb)

Additional file 5: 87 putative effectors in JWB phytoplasma genome assigned to Virulence Factor Database. (XLS 32 kb)

Additional file 6: 92 genes in JWB phytoplasma genome assigned to Pathogen Host Interactions Database. (XLS 46 kb)

## Abbreviations

ABC: ATP-binding cassette
BLASTP: Basic local alignment search tool-Protein
Ca: Candidatus
COG: Clusters of Orthologous Groups
GO: Gene Ontology
HGAP: Hierarchical genome-assembly process
JWB: Jujube witches’-broom
KEGG: Kyoto Encyclopedia of Genes and Genomes
NCBI: National Center for Biotechnology Information
NR: Non-Redundant Protein Database
ORFs: Ppen reading frames
PFGE: Pulsed-field gel electrophoresis
PHI: Pathogen Host Interactions
PMU: Potential mobile unit
VFDB: Virulence Factors of Pathogenic Bacteria
